# Epidemiological profile and diabetes control of Type 1 Diabetes Mellitus patients in Karbala Governorate, Iraq

**DOI:** 10.12688/f1000research.126561.1

**Published:** 2023-04-17

**Authors:** Abdul Razzaq Oleiwi Jasim, Noor Abdul Razzaq, Ali Thoulfikar A. Imeer, Rahem Mahdi Rahem, Abdul Amir H. Kadhum, Ahmed A. Al-Amiery

**Affiliations:** 1 College of Medicine, University of Al-Ameed, Karbala, Karbala, 5006, Iraq; 2 Karbala Health Directorate,, Ministry of Health, Karbala, Karbala, 5006, Iraq; 3 Dijlah University College, Baghdad, Baghdad Governorate, Iraq; 4 Chemical Engineering, Faculty of Engineering, Universiti Kebangsaan Malaysia, Bangi, Selangor, 43600, Malaysia

**Keywords:** Type1 diabetes, epidemiological characteristics, glycemic control, blood glucose

## Abstract

**Background**: Type1 Diabetes Mellitus is a common chronic diseases among children, and associated with morbidity, mortality, and enormous healthcare expenditures.

**Objectives**; to estimate the prevalence, incidence, and describe the epidemiological characteristics of Type 1 Diabetes Mellitus among children in Karbala governorate.

**Methods**: A cross-sectional study was conducted among all children who attended the main DM center in Karbala Teaching Hospital for Children, Public Clinics, and Primary Health Care centers. Data was collected by structured questionnaire and biochemical and anthropometric measurements. The statistical analysis data entry was conducted using Statistical Package for Social Sciences.

**Results**: Total number of Type 1 Diabetes Mellitus patients aged 0-15 years in Karbala in 2015 was 199; making 44.66/100 ^5^. Fifty-four patients were newly diagnosed patients making an incidence of 12.11/100 ^5^. There was nearly equal distribution among both sexes. Most of the cases were from urban areas and most of the patients had a Family history of Type 2 Diabetes Mellitus. Diabetic ketoacidosis was reported in 16.8% of the patients. Only 31.6% of patients had HbA1c < 7%. Half of the patients had a history of admission to the hospital for diabetes. Logistic regression analysis revealed that the only independent variables significantly correlated with poor glycemic control were lack of Self-Monitoring Blood Glucose, irregular visits to the Diabetic Center, and positive family history of diabetes.

**Conclusion**: Only one-third of T1DM children in Karbala city had controlled blood sugar. Lack of regular blood glucose monitoring and irregular contact with health care providers were the main determinants of uncontrolled blood sugar.

## Introduction

Type 1 diabetes mellitus (T1DM), is one of the most frequent chronic diseases among children and represents a public health challenge for many reasons globally the incidence, which represents about 10% of the diabetes cases all over the world. It is increasing especially among children below 15 years old and is increases 3% yearly worldwide. The first presentation of 10-70% of newly diagnosed children with DM1 is Diabetic Ketoacidosis (DKA). ^1^^,^^2^ The incidence rates differ in different populations, the lowest is observed in Venezuela and China while the highest in Sardinia and Finland. Regarding T1D incidence among Arab countries, several studies reported low incidence in Oman (2.54/100,000) and high incidence in Saudi Arabia (29/100,000). In general, the Arab countries show higher incidence and prevalence estimates in areas where DKA rates are high. ^3^ High DKA incidence results in increased hospital admission and emergency department (ED) visits and contributes to the high costs of care for children with T1DM. In Iraq, the prevalence of T1DM increased from 7.8 in 1995 to 14.2/100000 in 2000 and to 24.7/100000 in 2014 under 15 years old children. ^4^ Daily management of T1DM presents numerous challenges to achieving satisfactory metabolic control including multiple daily insulin injections, frequent blood glucose monitoring, frequent contact with medical professionals, and careful regulation of exercise, meal schedules, social status, and the educational level of the parents. ^4^^,^^5^ Studies in Iraq conducted on adolescents and children with Type 1 diabetes mellitus have shown that both patient and family education was associated with a lack in several emergency room visits and hospitalizations, a reduction in overall healthcare expense. Glycemic control in adolescents and children with type 1 diabetes mellitus in post-conflict in Iraq is low. They are poor in these diagnoses in pre-school age, adolescents, and obese children (23.8% of diabetic children had glycemic control), and it is much higher reported cases in Basrah, south of Iraq (10%). ^5^^,^^6^ While the Iraq Ministry of Health had some data on type 2 DM, there is scarce data on T1DM epidemiology. Availability of such data can help identify the burden of the problem and provide evidence that helps in the planning of health programs and allocation of financial and human resources, logistics, and treatment facilities. As the Karbala population is not different from the remaining Iraqi population, data from this study can be extrapolated and generalized to estimate corresponding figures representing Iraq. The aim of this study is to estimate the incidence and prevalence and to describe the epidemiological characteristics and the control status of T1DM among young patients in Karbala Governorate, Iraq.

## Methods

*Setting*: Karbala Governorate is located 100 Km to the south of Baghdad, the capital of Iraq with an estimated population of 1,122,400; 445,628 of them aged ≤15 years.

*Study design*: A cross-sectional study to measure the prevalence and identify basic socio-demographic data and complications. Also, a longitudinal incidence design was used to measure the incidence of the disease over one year.

*Study Population*: This study involved all T1DM patients who lived and registered in Karbala governorate, aged ≤15 years.

### Data Sources

Data of T1DM patients have been obtained from the following sources: 1) Karbala Teaching Hospital for Children where the main DM center is located, and the majority of young DM patients attended to seek medical support. 2) Public Clinics are distributed in all cities and towns throughout the country and they are the main health outlets where all patients with chronic diseases (including DM) receive their medications for a very subsidized cost. 3) Primary Health Care Centers (PH Care Cs) are distributed in all localities and provide all PHC services including school health services. Part of the activities of the school health program in these PHCs is to have a list of all students with chronic diseases (including DM) in the schools within the catchment areas of these PHCCs.

### Data Collection Tools

A data collection form was developed to compile data on all patients with T1DM from the registries of the diabetes center in Karbala Teaching hospital for Children, all the Popular Clinics, and all the PHCs Karbala governorate. These registries were useful in developing a list of patients currently diagnosed with T1DM, and the newly registered cases during 2013. Deceased patients during this year were first looked for at the Popular Clinics by identifying defaulters from the monthly medication supply lists. Information wars were verified through telephone communication with the families and school health programs in the PHCs. Clinical and epidemiological data were collected through the direct intervention of the patients using a structured questionnaire. The questionnaire was pre-tested on a sample of 10 patients from the target study population in the Diabetes Center. The measured parameters were the following anthropometric measures: Bodyweight, using a weighting digital scale (Seca) with light clothes without shoes and the weight was approximated to the nearest 100g), Height, using a portable measure (Seca), and height was approximated nearest 1cm. Body Mass Index (BMI) is calculated using the following equation: weight (in kilogram)/squared height (in meter). Moreover, measured fasting blood glucose (using Randox kit) using the colorimetric method), and Glycosylated hemoglobin (HbA1C) (using StandBio kit, using the spectrophotometric method).

### Ethical approval

Written informed consent was obtained from the caregiver of each participant before data collection. Confidentiality of the data was maintained throughout the study. Official approval was granted from the Karbala Directorate of Health.

*Statistical analysis*: Statistical Package for Social Sciences was used for data entry and statistical analysis. The frequency data were expressed in suitable tables, and graphs. The statistical association was tested using the chi-square test of independence and Fisher’s exact probability test when needed. Quantitative data were analyzed using an unpaired t-test. Logistic regression analysis was used to identify the significant independent determinants. A less than 0.05 of p-value of is considered significant statistically.

### Consent

Written informed consent for publication was obtained. The requirement for ethical approval was waived by the Medical Ethics Review Committee of the Academic Medical Center. Patients were treated according to guidelines of the Iraq Medical Association (KNMG). Medication was administered under Karbala Teaching Hospital and Primary Health Care Centers.

## Results

A total of 190 T1DM patients aged 0-15 years were identified making a prevalence of 44.66/10 ^5^. Fifty-four patients were newly diagnosed patients during 2013, making an incidence of 12.11/10 ^5^. The Mean age was 8.8 (±3.6) years; 55.8% were more than eight years old and 14.2% were less than five years. About 53% were females and 68% were urban residents. Breastfeeding was reported by 93.2% of the patients; 77% of them continued breastfeeding for more than six months. Positive family history of T1DM and exposure to significant stressful life events were reported in 20.5% and 21.5%, respectively. As shown in Table 1, more than one-fifth (23%) of the patients presented with diabetic ketoacidosis (DKA) as a first manifestation of the disease, and around 22% had the disease for more than six years. Only one-third of the patients (31.6%) had controlled diabetes as reflected by HBA1c; 28% had a history of severe acute complications (hypoglycemia or DKA) and 51.5% required admission either to the ER or the inpatient ward.

**Table 1.** Distribution of T1DM patients by clinical characteristics.

Variables			No. (190)	Percent
Clinical presentation at diagnosis	Classic symptoms		138	72.6
	DKA		43	22.6
	Accidental		9	4.7
Duration of the disease in years	<6		148	77.9
	> 6		42	22.1
HbA1C%	< 7%		60	31.6
	7-8		32	16.8
	> 8%		98	51.6
Acute complications	No		139	73.2
	DKA		32	16.8
	Hypoglycemia		19	10
Hospitalization	Yes	Emergency Room	17	8.9
		Word	71	41.6
	No		94	49.5

To identify the determinants of the control status of the disease among the young T1DM patients, we compared patients with controlled diabetes as reflected by normal HBA1c, with the group of uncontrolled disease by several sociodemographic and clinical characteristics and certain behavioral factors as demonstrated in Tables 2 and 3. The proportion of controlled disease was significantly higher among children aged ≤8 years (P=0.008), with better mothers’ and fathers’ education (P<0.001), employed fathers (P=0.017), and children with a duration of disease <6 years (P=0.034). ( Table 2). Also, controlled diabetes was significantly higher among patients with regular attendance at the diabetes center (P< 0.001), blood glucose self-monitoring (P< 0.001), adherence to insulin treatment, and controlled dietary intake by the parents (P< 0.001). ( Table 3).

**Table 2.** Distribution of the study participants by blood glucose control status.

Variables		P-Value				P-Value
		Controlled N0. %		Uncontrolled No. %		
Gender	Male	29	32.2	61	67.8	0.610
	Female	31	31	69	69.0	
Age in years	≤8	35	41.7	49	58.3	0.008
	>8	25	23.6	81	76.4	
Residence	Urban	47	36.2	83	63.8	0.032
	Rural	13	21.7	47	78.3	
Parents Marital status	Living together	56	31.1	124	68.9	0.392
	Divorced/Widowed	4	40.0	6	60.0	
Mother’s Educational level	Illiterate/Primary	25	21.2	93	78.8	<0.001
	Secondary	23	41.1	33	58.9	
	University	12	75.0	4	25.0	
Father’s Educational level	Illiterate/Primary	14	16.7	70	83.3	<0.001
	Secondary	19	30.6	43	69.4	
	University	27	61.4	17	38.6	
Father’s Employment status	Jobless (or dead)	0	0.0	6	100.0	0.000
	Employed	60	48.4	124	42.6	
Mother’s employment status	Employed	9	60.0	6	40.0	0.017
	Housewife	51	29.1	124	70.9	
Family history of diabetes	Positive	27	22.7	92	77.3	<0.001
	Negative	33	46.5	38	53.5	
Duration of the disease	<6 years	52	35.1	96	64.9	0.034
	>6 years	8	19.0	34	81	
Co-morbid diseases	Negative	44	33.6	87	66.4	0.237
	Positive	16	27.1	43	72.9	

**Table 3.** Distribution of T1DM patients by certain clinical and behavioral factors.

		B. Glucose control status				
		Controlled		Uncontrolled		
		No	%	No	%	
Visiting diabetes center	Regular	58	69.9	25	30.1	.000
	Irregular	2	1.9	105	98.1	
Monitoring blood glucose	Regular	54	66.7	27	33.3	.000
	Irregular	6	5.5	103	94.5	
Adherent to insulin	No	6	10.7	50	89.3	.000
	Yes	54	40.3	80	59.7	
Controlled physical activity	No	36	31	80	69	.840
	Yes	24	32.4	50	67.6	
Controlled Diet	No	2	2.7	73	97.3	.000
	Yes	58	50.4	57	49.6	
Bodyweight level	Underweight	8	22.2	28	77.8	.134
	Healthy	50	33.3	100	66.7	
	Overweight	2	50	2	50	

**Table 4.** Logistic regression analysis (OR with 95% CI) for factors related to the glycemic state of children in Karbala city, Iraq.

Variables	OR	95% CI		
		Lower	Upper	P-Value
**Sociodemographic characteristic**				
Residence	3.67	.72	18.55	.117
Age	1.54	.58	4.09	.383
Educational level of the fathers	.51	.20	1.27	.148
Educational level of the mothers	2.27	.80	6.47	.126
**Management Behaviors**				
Irregular Monitoring of blood glucose	10.41	2.24	47.62	**.001**
Irregular visits to DM center	31.05	5.62	171.32	**.001**
Diet as recommended	.12	.01	1.06	**.057**
Cold storage of insulin	.07	.01	2.79	.159
**History of the disease**				
Family history of diabetes	1.86	1.01	3.41	.046
Duration of the disease	.55	.18	1.62	.275

Logistic regression analysis was applied considering the poorly controlled status as the dependent variable and all the variables that were found significant in the binary analysis were included as the independent variables in the model. Three factors were found statistically significant: self-monitoring of blood glucose (OR:10.41, 95% CI:2.24-47.62) irregular attendance to the diabetes center (OR:5.62, 95%CI: 5.62-171.32), and family history with positive DM (OR:1.86; 95% CI:1.01-3.41).

## Discussion

### Incidence and prevalence

In the current study all the patients were 199, among them there was 54 new patients, 190 patients (95.47%) participated in this study, and the incidence rate (12.11/100,000) was higher as compared to another study in Basrah city, Southern Iraq, (7.4 per 100,000 (95% CI, 7.1-8.1). ^5^ Japan (2.2/100,000), Iran (3.7/100,000), and lower compared to, United Kingdom (26/100,000) ^2^ Saudi Arabia (33.5/100,000). ^7^ While prevalence (44.65/100,000) was higher compared to study in South-Eastern Nigerian school children aged 5–17 years. (33/100,000), ^8^ and lower than Saudi-Arabian (109.5/100,000). ^9^

### Sociodemographic characteristic

Most of our patients aged more than 8 years, most of them from rural areas, Sex nearly equal distribution with only slight female predominance. A study from Al-Madinah/Saudi Arabia has reported a higher incidence for the 10–12-year age group than in younger children. Furthermore, they have reported a higher incidence of T1DM in girls than in boys and most patients from urban areas, ^7^^,^^10^ in the current study most of the mothers were housewives, with low educational levels, in constituent with another study in Egypt in which 85% of the patients with not work mothers and about two-thirds of them had low educational level. ^11^

### Rate control

Achieving the ideal blood glucose level is very difficult for many patients with diabetes, therefore, in the current study, the rate of optimal glycemic control (HbA1C <7%) represented around 31.6% of the patients comprised in other studies in Kenya (28%), ^12^ Saudi Arabia (31.2%), Egypt (60%) and two-thirds of Italian children with T1DM have HbA1c>8% despite a regionalized centers, free access to appropriate diabetes care, multidisciplinary team approach, frequent blood glucose monitoring, education and multiple insulin injections. ^13^

### Gender, Residence, Age of patients, and marital status of their parents

Good glycemic control is associated with age < 8 years, urban residence, not affected by gender, reflecting the same complex the social and economic environment in which both genders are living, another study in Saudi Arabia confirmed the same result. ^13^ other studies found better control associated with older age ^14^ and a few studies shows that the mean of HbA1C in girls was significantly higher than in boys. ^15^ another study from the UK showed a linear association between residence and glycemic control in type 1 diabetes ^16^ and Our results reflected a very low incidence of divorce in the study group, so there was no significant correlation between glycemic state and marital status of the parents.

### Self-Monitoring of Blood Glucose (SMBG) & Regularity of Visits to Diabetic Center

In the current study, good blood glucose monitoring (BGM), adherence, and regularity of clinic attendance were significantly associated with better HbA1c, in agreement with several studies. ^14^^,^^17^ The clinic visits frequently is recommended to allow for better frequent adjustment of insulin regimens, and an increases number of opportunity for motivation and education.

### Education, Occupation of the parents, and family history

More knowledgeable parents on diabetes with better education are able to cope more effectively and maintain better glycemic control of their diabetic children, results in other studies showed a significant relationship between low education of parents and poor glycemic control, ^11^^,^^18^ majority of our patients had housewife mothers and most of their fathers were self-employed, such type of life does not permit optimal looking after the diabetic Childs, then consequently it turns to uncontrolled status of HbA1C levels, we found that positive family history of T2DM was more frequent than type 1 or both, other study found that only 10–15% of the patients have a first- or second-degree relative with T1DM, ^1^ while family history of type 1 diabetes mellitus is highly prevalent among other studied patients. ^19^

### Duration, adherence to a healthy diet and insulin

Our study is in agreement with many other studies, which concluded a significant correlation between the level of HbA1c and diabetes duration (poor glycemic control in patients with a duration of 6 years and more), adherence to dietary management, and insulin. ^13^^–^^14^^,^^20^

### Acute complications and Hospitalization

16.8% of the patients had DKA, 8.9% had hypoglycemia, in 2013, and by the ranking of countries according to the frequency of DKA, the current study demonstrated that the result was lower than the frequencies were observed in Saudi Arabia (44.9%), the United Arab Emirates (80%), Hungary (23%) and Finland (22%). The lowest frequencies for DKA presentation of T1DM is reported in Sweden (14%). ^9^ Initially, a classical type of presentation was found in about two-thirds of our patients, In comparison to a study conducted in Brazil, approximately 20% of patients with previously undiagnosed DM1 initially presented with DKA ^21^ while another study found 68% of children had DKA. ^22^ About half of diabetic patients are admitted to the hospital, in Sweden children newly diagnosed with type 1 diabetes mellitus are admitted to the hospital for metabolic stabilization and training, even if they are not acutely ill, diabetic ketoacidosis and hypoglycemia often lead to an emergency department (ED) visit and hospital admission. In another study, in Sudan, most of the children (81%) had a history of hospital admission with DKA. ^23^ Mortality in patients with childhood-onset type 1 diabetes in the current study was zero, during the time of the study. No chronic complication was found in all patients. Despite the developments in clinical care in recent years, the mortality risk for people developing type 1 diabetes in childhood remains high in young adult life before the onset of chronic complications. ^24^^,^^25^

## Conclusion

Poor glycemic control was in around two-thirds of the patients and about 25% of them had acute complications and problems in school achievement. About half of the patients were admitted to the hospital. The most important co-varieties of the uncontrolled glycemic state of the patients were older age children, low educational level of the parents, and rural residence factors. SMBG, irregular visits to the Diabetic Center, and family history of diabetes were significant predictors of poor rate control.

### Recommendations

The result of this study suggests that the pediatrician and endocrinologists must be critically assess the care required to these group with considering any new approaches to improve controlling their glycemic level. Special attention should be taken to develop a public health intervention strategy to educate the population and increases their awareness about the risk factors of diabetes complications. Stress on the practice of regular visit to the care Center for the proper monitoring of the disease and preventing any complication. Our finding is confirmed the need to develop a national registry for T1DM and the need for further multicenter epidemiological research studies covering the entire country to define the nationwide T1DM incidence for the related health data in Iraq.

## Author contributions

A.R.O.J., N.A.R., A.T.A.I., R.M.R., and A.A.H.K., were responsible for the design of the treatment protocol and done all the tests. A.A.A-A. draft the manuscript. All authors were involved in the revision of the draft manuscript and have agreed to the final content.

## Data Availability

Zenodo. Epidemiological profile and diabetes control of Type 1 Diabetes Mellitus patients in Karbala Governorate, Iraq. DOI: 10.5281/zenodo.7380413. ^26^ Figshare. dx.doi.org/10.6084/m9.figshare.6025748. DOI: https://doi.org/10.6084/m9.figshare.6025748.v1. ^27^ These projects contain the following data:
- Source data for the figures of the article - Statistical results - Data are available under the terms of the Creative Commons Attribution 4.0 International license (CC-BY 4.0).
