# Supplementary material for: Epidemiological profile and diabetes control of Type 1 Diabetes Mellitus patients in Karbala Governorate, Iraq
Source: F1000Res. 2023 Apr 17;12:409. [Version 1] doi: 10.12688/f1000research.126561.1 (PMC11409437; doi:10.12688/f1000research.126561.1)
Supplement: Peer review history

# Reviewer report: Reviewer response for version 1

**DOI:** 10.5256/f1000research.138985.r311405

**Published:** 2024-09-12

**Authors:** Charlotte Verroken

**Affiliations:** 1 Dept. of Endocrinology,, Ghent University Hospital,, Ghent, Belgium

The article is well-structured.

 The most important concern is that the methodology needs to be more clearly described. How and in which time frame were participants recruited (were all T1DM patients invited or those that visited the clinics within a certain timeframe)? How high was the participation rate? Who performed the anthropometrics (the research team or was this information retrieved from the patient files)? Were lab analyses specifically performed for the study or also retrieved from patient files?

 In addition, the article might benefit from language editing.

Is the work clearly and accurately presented and does it cite the current literature?

Partly

If applicable, is the statistical analysis and its interpretation appropriate?

Yes

Are all the source data underlying the results available to ensure full reproducibility?

No source data required

Is the study design appropriate and is the work technically sound?

Yes

Are the conclusions drawn adequately supported by the results?

Yes

Are sufficient details of methods and analysis provided to allow replication by others?

No

Reviewer Expertise:

NA

I confirm that I have read this submission and believe that I have an appropriate level of expertise to confirm that it is of an acceptable scientific standard, however I have significant reservations, as outlined above.

# Reviewer report: Reviewer response for version 1

**DOI:** 10.5256/f1000research.138985.r311391

**Published:** 2024-08-28

**Authors:** Kamarudin Ahmad

**Affiliations:** 1 Miri Regional Pharmacy Office, Miri, Sarawak,, Malaysia

Abstract

 The abstract of the manuscript provides a concise overview of the study. However, it could benefit from a stronger emphasis on the methods section. Detailing the study design, sampling strategy, and key statistical approaches in the abstract would offer readers a clearer understanding of how the research was conducted. This would also enhance the transparency and reproducibility of the study.

**Suggestions:**

- **Include Methodological Details:** Briefly mention the logistic regression analysis and any specific inclusion/exclusion criteria used to select participants.
- **Highlight Key Findings:** Summarize the most significant results to give a snapshot of the study’s outcomes.

Introduction

 The introduction currently lacks a smooth and directive flow. It would be more effective to explicitly discuss the problem being addressed by the study, using a funnel approach that starts broad and narrows down to the specific research aims. The introduction should also incorporate a detailed explanation of the current situation, supported by relevant literature, and link these to the study's objectives.

 Additionally, the introduction should explain the factors influencing the variables included in the logistic regression analysis, supported by evidence from the literature. This will help set the stage for the analysis and interpretation of results.

**Suggestions:**

- **Improve Structural Flow:** Revise the introduction to ensure each section logically leads to the next, connecting background information to research questions and objectives.
- **Contextualize Variables:** Explain the factors influencing the variables in the logistic regression model with supporting literature.
- **Consistency in Terminology:** To avoid confusion, use terms like "glycemic control," "blood sugar control," and "HbA1c levels" consistently throughout the introduction.

Methods

 The methods section requires reordering, with the statistical analysis discussed before the ethical approval. Additionally, not all variables included in the logistic regression analysis are explained in detail. A thorough explanation of each variable, including how they were measured and why they were included in the regression model, is crucial for the reader's understanding.

**Suggestions:**

- **Reorder Sections:** Present the statistical analysis methods before discussing ethical approval to follow a logical sequence.
- **Detailed Variable Explanation:** Provide a clear rationale for including each variable in the logistic regression model and describe how each was measured.
- **Clarity in Ethical Considerations:** Clearly outline how ethical considerations influenced the study design, particularly in a sensitive context like pediatric diabetes. This highlights the researchers' commitment to ethical rigour.

Results

 There is evidence of selective outcome reporting in the results section. Specifically, not all results from the logistic regression analysis are displayed. This selective reporting could undermine the study's credibility and the robustness of its conclusions.

**Suggestions:**

- **Comprehensive Reporting:** Ensure that all results from the logistic regression analysis are presented, including both significant and non-significant findings. This transparency strengthens the study's validity.
- **Data Visualization:** Incorporate more visual elements such as graphs or tables to summarize key findings, particularly from the logistic regression analysis. Visual aids help readers quickly grasp complex data and understand the relationships between variables.

Discussion

 The discussion section inappropriately includes results that should be confined to the results section. The discussion should focus on interpreting the findings, explaining the implications of the results, and discussing them in the context of existing literature. Moreover, there is a need to explain why certain variables were significant in the logistic regression analysis, supported by relevant evidence.

**Suggestions:**

- **Restructure Content:** Avoid presenting new results in the discussion. Focus on interpreting the results already presented and discussing their implications.
- **Justify Findings:** Explain why certain variables were significant in the logistic regression analysis, supported by relevant literature.
- **Highlight Study’s Limitations:** Include a detailed discussion of the study's limitations, such as potential biases (e.g., selection bias or confounding factors), and how these were addressed or could be addressed in future research. This transparency strengthens the study’s credibility.
- **Future Research Directions:** Expand on the implications of the findings for future research. For example, suggest exploring significant variables further in longitudinal studies or different populations to validate the findings.
- **Impact on Clinical Practice and Policy:** Discuss how the findings could influence clinical practice or public health policy, especially in the context of pediatric diabetes management. Explore how regular monitoring and family education could be better integrated into public health initiatives.

Conclusion

 The conclusion currently makes statements not fully supported by the results presented. The conclusion must be directly backed by the study's findings, without overgeneralizing or making unsupported claims.

**Suggestions:**

- **Align with Results:** Ensure the conclusion accurately reflects the study's results. Avoid introducing new information or making extrapolations beyond the data presented.
- **Clear and Concise:** Summarize the key findings and their implications succinctly, reinforcing the study’s contributions without overstating them.

Additional Suggestions

1. **Consistency in Terminology:**
  - Ensure that the terminology is consistent throughout the manuscript. To avoid confusion, terms like "glycemic control," "blood sugar control," and "HbA1c levels" should be used uniformly.
2. **Highlighting the Study’s Limitations:**
  - Acknowledge potential biases, such as selection bias or confounding factors, and discuss how these were addressed or could be mitigated in future research. This enhances the study’s credibility.
3. **Future Research Directions:**
  - Encourage the authors to outline specific areas for future research based on their findings, such as conducting longitudinal studies or expanding the research to different populations to validate and extend the results.
4. **Impact on Clinical Practice and Policy:**
  - Suggest ways the findings could inform clinical guidelines or public health policies, particularly in improving pediatric diabetes management through regular monitoring and education initiatives.
5. **Data Visualization:**
  - I recommend using additional visual elements like graphs, charts, or tables to effectively present complex data and highlight key relationships identified in the logistic regression analysis.
6. **Clarity and Consistency:**
  - Ensure that all manuscript sections use consistent terminology and maintain a clear, logical flow to enhance readability and comprehension.

Overall Structure and Presentation

- **Consistency and Clarity:** Maintain consistent terminology and ensure each section transitions smoothly to the next.
- **Detailed Reporting:** Provide sufficient detail in the methods and results sections to allow replication and full understanding by readers.
- **Transparent Reporting:** To enhance the study's transparency and reliability, present all findings comprehensively, including non-significant results.
- **Comprehensive Discussion:** Focus on interpreting results, addressing limitations, and outlining future research directions without introducing new data.

Is the work clearly and accurately presented and does it cite the current literature?

No

If applicable, is the statistical analysis and its interpretation appropriate?

Partly

Are all the source data underlying the results available to ensure full reproducibility?

Yes

Is the study design appropriate and is the work technically sound?

Partly

Are the conclusions drawn adequately supported by the results?

Partly

Are sufficient details of methods and analysis provided to allow replication by others?

Partly

Reviewer Expertise:

My research is dedicated to advancing the safety and effectiveness of medication management within community and clinical settings. It emphasizes reducing prescribing errors through pharmacist-led interventions and other systematic approaches. A significant aspect of this research involves exploring the side effects of widely used medications, such as metformin in Type 2 Diabetes Mellitus, by conducting comprehensive reviews of existing studies.Collaborating with healthcare professionals aims to translate research findings into practical applications that improve patient care. Research interests extend to international collaborations, particularly between Malaysia and Thailand, focusing on drug security, medicines policy, and primary care pharmacy. The ultimate goal is to enhance patient outcomes by ensuring safer and more effective use of medications.

I confirm that I have read this submission and believe that I have an appropriate level of expertise to confirm that it is of an acceptable scientific standard, however I have significant reservations, as outlined above.

# Reviewer report: Reviewer response for version 1

**DOI:** 10.5256/f1000research.138985.r180900

**Published:** 2023-06-30

**Authors:** Kalyani Pathak

**Affiliations:** 1 Dibrugarh University, Dibrugarh, Assam, India

This paper provides valuable insights into the prevalence and epidemiological characteristics of Type 1 Diabetes Mellitus in children in Karbala. However, it would benefit from more clarity regarding the methodology. The use of a structured questionnaire, biochemical, and anthropometric measurements are mentioned, but specific details are missing. For example, it would be helpful to know the exact questions asked in the questionnaire and the nature of the biochemical measurements. Also, it would be interesting to understand how the authors controlled for potential confounding factors.

 The manuscript is well-structured and provides important information on Type 1 Diabetes Mellitus in children. However, it would be beneficial to provide more context on the implications of the findings. The authors mention that only 31.6% of patients had HbA1c < 7%, but the significance of this finding for the patient population or the health care system is not discussed. It would be useful to elaborate on what these results mean for future research, public health policies, or clinical practices in Karbala.

Is the work clearly and accurately presented and does it cite the current literature?

Yes

If applicable, is the statistical analysis and its interpretation appropriate?

I cannot comment. A qualified statistician is required.

Are all the source data underlying the results available to ensure full reproducibility?

Yes

Is the study design appropriate and is the work technically sound?

Yes

Are the conclusions drawn adequately supported by the results?

Yes

Are sufficient details of methods and analysis provided to allow replication by others?

Yes

Reviewer Expertise:

Diabetes, Traditional medicine

I confirm that I have read this submission and believe that I have an appropriate level of expertise to confirm that it is of an acceptable scientific standard.
